# Novel method to decrease the exposure time of the extraction string of the ureteral stent and its efficiency and safety verification in the clinic

**DOI:** 10.1038/s41598-021-01821-2

**Published:** 2021-11-16

**Authors:** WenGang Hu, YaJun Song, Yang Li, YueHua Li, Jiao Mu, Xiao Zhong, YiRong Chen, RongHua Wu, Ya Xiao, ChiBing Huang

**Affiliations:** 1 Urology Department, The 2nd Affiliated Hospital, The Army Medical University of China, No. 183, XinQiao Street, ShaPingBa District, Chongqing, 400037 People’s Republic of China; 2 State Key Laboratory of Trauma, Burns and Combined Injury, Institute of Burn Research, The First Affiliated Hospital of Army Medical University, Chongqing Key Laboratory for Disease Proteomics, Chongqing, 400038 People’s Republic of China; 3 Nephrology and Urology Department, The Affiliated University-Town Hospital, ChongQing Medical University, Chongqing, 401331 People’s Republic of China; 4 Urology Department, The Three Gorges Affiliated Hospital, ChongQing University, Chongqing, 404000 People’s Republic of China; 5 Urology Department, The 3rd Affiliated Hospital, The Army Medical University of China, Chongqing, 400042 People’s Republic of China

**Keywords:** Health care, Urology

## Abstract

Ureteral stent removal by an extraction string is advantageous. However, the increased risk of complications attributed to the continuous exposure of the string outside the urethra must be managed. This paper introduces a method to decrease the exposure time, and conducts a retrospective study to verify its efficiency and safety. A total of 231 male patients undergoing routine ureteroscopy (URS) were included, and all of them accepted indwelling ureteral stents with strings. Among them, 123 patients (Normal-S group) underwent the normal method to determine the length of string (L_string_), which was shortened to 4 cm (cm) past the urethral meatus; 108 patients (Novel-S group) underwent the novel method (L_string_ = L_urethra_ + 2 cm), the length of urethra (L_urethra_) was measured during ureteroscopy by ureteroscope body. The demographic characteristics, stent indwelling and removal-related variables, complications, and medical costs in each group were recorded. There was no significant difference in demographic characteristics, the rate of UTI, the operative duration of URS, or the VAS pain scores for stent removal between the 2 groups. For the Novel-S group, the stent dwelling time was longer, the self-rated discomfort and symptom, the stent dislodgement rate, the numbers of clinic or emergency visits and the overall medical cost post operation was lower in comparison with the Normal-S group, while the rate of removal of stents by hand was lower, the time for removing ureteral stents was longer. This novel method improved stenting comfort, avoided ureteral stent dislodgement, decreased complications, and lowered medical costs, it was safe and reliable and merits widespread application.

## Introduction

Ureteral stenting represents a simple and effective drainage method to preserve renal function. Infection, stones, strictures external compression of the ureter and surgical procedures, such as ureteroscopies, render stenting necessary^1^. Decreasing stent-related complications and improving the patients’ comfort degree and tolerability have been popular research topics in recent years. The postoperative complications of indwelling ureteral stents are mainly derived from two aspects. One is in situ-related ureteral problems caused by its characteristics of foreign material, which can be alleviated by optimizing the material for fabricating stents^1–3^. The other is related to ureteral stent removal^4^. Traditional removal by cystoscopy is an invasive procedure that causes urethral injury and augments the pain of the patient, especially for males^5^. Although removal by a flexible cystoscope can decrease the injury to some degree, it is also time consuming and laborious, and more importantly, the pain and injury caused by the operation are also considerable^6,7^. Therefore, to solve this problem, many studies have been conducted, including with biodegradable ureteral stents, which are not necessary to remove^8^, and ureteral stents with extraction strings, which can be removed by hand^4,5,9,10^.

Biodegradable ureteral stents, which need not be removed, can be degraded or dissolved by urine, and their main advantage is that secondary intervention becomes unnecessary, decreasing the economic burden on the medical system as another advantage^11^. However, the controllability of degradation and uneven migrating fragments from stent degradation still represent an issue that must be overcome^1,3^. Stent with extraction string was first described by Siegel et al. in 1986^12^. It can be withdrawn by pulling the string with hand at the scheduled time. This method was convenient because it obviated an invasive cystoscopic procedure. It has been widely applied in many medical institutions worldwide. Its safety and efficiency have been demonstrated. Recent systematic studies have confirmed that this method provides significantly less pain than cystoscopy without increasing the risk of urinary tract infection or stent-related urinary symptoms, with the exception of increasing the risk of stent dislodgement^4,5,7,10,13^.

Upon analyzing the reasons for the increased risk of dislodgement, continuous exposure of the extraction string outside the urethra was discovered to be the main reason; tugging at the stent string when bathing or voiding can incidentally lead to accidental removal^10,13^. Theoretically, as the exposure time increases, the risk of dislodgement increases. In the 5 years of implementing this method in our urology department, we dedicated ourselves to exploring a way to diminish the exposure time of the string. Occasionally, it was found that if the length of the string was appropriate for males, it was only excluded from the urethral meatus during urination, which significantly decreased the exposure time. Therefore, 5 years ago, we designed a method to determine the length of the string required and then implemented it 3 years ago, which accomplished the purpose referred to above in most cases. In this study, the method is described, and a retrospective study was conducted to verify its efficiency and safety compared with the normal removal method by extraction string.

## Methods

### Patients

This study was approved by the Ethics Review Board of the Second Affiliated Hospital of the Army Medical University of China. All methods were performed in accordance with the Declaration of Helsinki and other relevant guidelines and regulations. Male patients undergoing routine URS for unilateral ureteral stones and having an ureteral stent inserted after URS in the hospital between January 2016 and December 2019 were included. All participants accepted indwelling ureteral stent with a string, and informed consents were obtained from them.

The exclusion criteria were the following: (1) female sex; (2) juvenile status (younger than 16); (3) coexisting noncalculous disease (e.g., malignant obstruction, renal insufficiency, congenital anomaly of urinary tract, or ureteral stricture); (4) solitary kidney; (5) patients undergoing bilateral URS; (6) patients with hypoimmunity (taking immunosuppressive or chemotherapeutic drugs); (7) complicated URS requiring long-term stent placement (> 7 days); and (8) patients without follow-up data.

### Surgical procedure and study design

All of the operational procedures were performed in the urology department by 3 credentialed surgeons, who participated in URS and stent removal. The common surgical procedure mainly includes 5 steps: (1) inserting ureteroscope for ureteral examination; (2) laser lithotripsy after locating the ureteral calculus; (3) retaining guide wire and withdrawing the ureteroscope after finishing the lithotripsy; (4) indwelling ureteral stent along the wire; (5) guide wire removal and urethral catheterization. The urethral catheter was pulled out in 24 h upon indwelling. All patients were discharged 2 days after the URS, prophylactic antibiotics were administered for 2 days. All of them were reminded to be cautious regarding the string to prevent inadvertent extraction, and they were informed to return to the hospital for removal of the ureteral stent on the eighth day after surgery; however, 1–2 days in advance was allowed due to discomfort in urination, and a 3- to 4-day delay was also allowed with no syndrome or discomfort or if the date was not convenient.

Normal-S group (From the year of 2016–2017).

Ureteral stent indwelling: In this group, a 6F stent from Cook Medical was inserted via a retrograde approach after completing the URS. The stent string was a 4–0 silk suture passed through the venting side hole of the stent with a quadruple knot to prevent slippage and distraction^14^, and its length was shortened to several centimeters (4 cm long) past the urethral meatus^4,9,15^. The string was freely placed, it was not affixed to the patient in any fashion.

Ureteral stent withdrawal: a doctor pulled the string discharged to the external urethral orifice by continuous and gentle force (Supplementary Information 1).

#### Novel-S group (From the year of 2018–2019)

**Figure 1:**
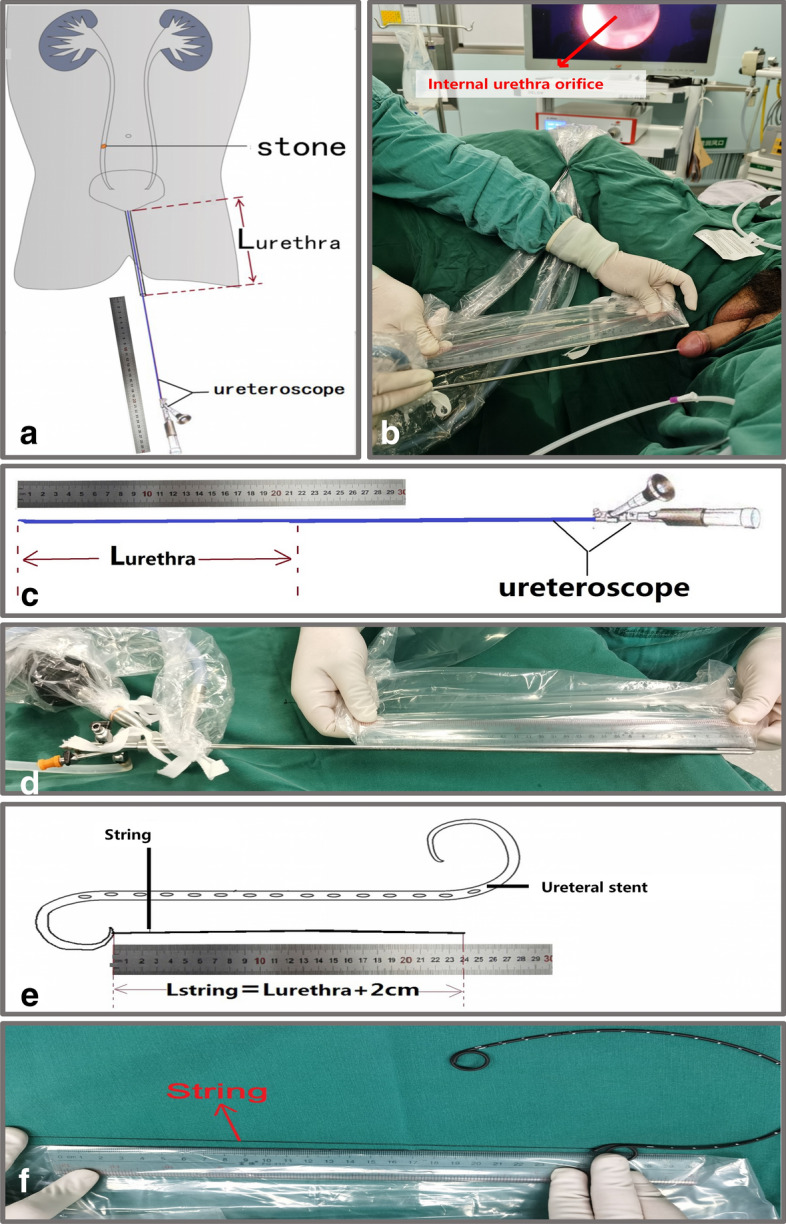
Diagrammatic sketch/surgery photo of marking Lurethra (**a**,**b**), measuring Lurethra (**c**,**d**) and determining Lstring (**e**,**f**).

Ureteral stent indwelling: During the URS, a ureteroscope was inserted into the urethra from the external urethral orifice; when its front end reached the internal urethral orifice, the inserting length of the ureteroscope was marked with the penis in the natural state (Fig. 1a,b), and this length was deemed to be the length of the urethra (L_urethra_), measured outside the body (Fig. 1c,d). The stent string was manipulated in the same way as in the Normal-S group, but its total length (L_string_) was L_urethra_ + 2 cm (Fig. 1e,f), different from the Normal-S group.

Ureteral stent withdrawal: (1) The string was outside the urinary meatus all the time as in the Normal-S group: the removal method was same as in the Normal-S group. (2) The string was discharged outside the urinary meatus only during urination (Supplementary Information 2): The doctor grasped the string during urination and then removed the stent as in the Normal-S group. If the string could not be discharged through the urinary meatus (Supplementary Information 3), then ureteroscopy into the anterior urethra was conducted to remove the stent (Supplementary Information 4), without the necessity of inserting an ureteroscope into the posterior urethra or bladder; however, normal ureteroscopy into the bladder was conducted if the string was undischarged to the anterior urethra.

### Outcome assessment

Demographic and patient characteristics were registered, including sex, age and BMI.

Preoperative and operative variables were recorded, including the stone side, stone location (upper, middle, lower), and stone size. preoperative stent, first ureteral stone and operative duration.

Complications related to the stent string were evaluated with regard to stent dislodgement (defined as the inadvertent removal of the stent after indwelling), urinary tract infection (UTI: it was diagnosed according to the American National Healthcare Safety Network standardized surveillance criteria^16^), self-rated discomfort and symptom (A Likert scale from 1 point (best) to 5 points (worst) to grade them respectively), clinic or emergency visits, retained stents, and the medical expense of managing the complications.

Stent indwelling and removal-related data were collected, including the operative duration of URS, success rate of stent removal by hand, stent dwell time, time to remove the stent and visual analog scale (VAS) pain score at stent removal and 1 h after removal.

The medical expense postoperatively was recorded in terms of the medical cost for dislodgement, UTI, clinic or emergency visits, the cost of stent removal, and the overall cost postoperatively.

### Statistics

Statistical analyses were performed using SPSS software, version 18.0 (SPSS Inc., Chicago, IL, USA). Numerical data were compared using the independent-samples t-test or nonparametric test. Categorical data were analyzed using the chi-square test. Statistical significance was accepted at p < 0.05.

### Ethical declarations

This study was approved by the Ethics Review Board of XinQiao Hospital (the Second Affiliated Hospital of the Army Medical University of China). The approval number is 2015002-11.

### Consent to participate

All patients consented to participate in the study.

### Consent for publication

All patients included and all authors consented for publication of the paper.

## Results

A total of 231 patients were included in the study. There were no significant differences in demographic characteristics between the two groups (Table 1).

**Table 1:** Demographics of the two groups and complications related to stent string.

	Normal string-stent group Normal-S group	Novel string-stent group Novel-S group	P-Value
Mean age, years(SD)	43.4 (14.6)	46.5 (14.9)	0.115
Mean BMI, kg/m^2^(SD)	24.5 (2.5)	23.9 (2.6)	0.088
**Stone side, n (%)**			0.327
Left	58 (47.2)	44 (40.7)	
Right	65 (52.8)	64 (59.3)	
**Stone location, n (%)**			0.445
Upper	14 (11.4)	9 (8.3)	
Mid	63 (51.2)	64 (59.3)	
Lower	46 (37.4)	35 (32.4)	
Mean stone size, mm (SD)	8.8 (1.8)	8.5 (1.8)	0.130
Preoperative stent, n (%)	12 (9.8)	8 (7.4)	0.527
**First ureteral stone or not, n (%)**			0.530
First	69 (56.1)	65 (60.2)	
Not first	54 (43.9)	43 (39.8)	
Stent dislodgement, n (%)	9 (7.3)	0 (0)	0.004
UTI, n (%)	8 (6.5)	5 (4.6)	0.537
Clinic or emergency visits, n (%)	31 (25.2)	9 (8.3)	0.001

No differences were observed in the rate of UTI, the operative duration of URS, or the VAS pain scores at stent removal and 1 h after removal for the two groups (p > 0.05) (Tables 1 and 2).

**Table 2:** Stent indwelling/removal-related data and medical expense.

	Normal string-stent group Normal-S group	Novel string-stent group Novel-S group	P-Value
Mean operative duration of URS, min (SD)	31.6 (9.3)	33.0 (10.9)	0.302
Mean stent dwell time, days (SD)	7.2 (0.9)	10.7 (1.7)	0.001
**Self-rated discomfort and symptom, scores (SD)**			
Discomfort in urethra	2.6 (1.6)	1.7 (1.1)	0.001
Urine spattering symptom	2.4 (1.0)	1.4 (0.7)	0.001
Stent removal, n (%)	123 (100)	108 (100)	/
By the string (during urinating/not urinating)	123 (100) (0/123)	101 (93.5) (92/9)	0.004
By ureteroscopy in the anterior urethra	0 (0)	7 (6.5)	/
Mean time for removing the stent, s (range)	26.7 (18–35)	65.3 (19–482)	0.001
Mean VAS pain score in stent removal, scores (SD)	1.9 (0.8)	2.0 (0.9)	0.443
Mean VAS pain score one hour after stent removal, scores (SD)	0.5 (0.6)	0.6 (0.7)	0.114
Mean medical cost for dislodgement, UTI, clinic or emergency visits, CY (range)	55.8 (0–373)	17.3 (0–258)	0.001
Mean cost for stent removal, CY (range)	0 (0)	10.4 (0–125)	0.001
Mean overall cost of postoperation, CY (range)	55.8 (0–373)	27.7 (0–258)	0.047

The self-rated discomfort and symptom, the stent dislodgement in the Normal-S group was higher than that in the Novel-S group (2.6 vs 1.7, p = 0.001; 2.4 vs 1.4, p = 0.001; 7.3% vs 0%, p = 0.004 respectively, Tables 1 and 2), 9 patients in the Normal-S group had experienced stent slippage, among of them, 4 patients reported accidental dislodgement during taking off pants, 5 ones in bathing. The clinic or emergency visits of the Normal-S group were also higher: 25.2% vs 8.3% (Table 1). However, the stent dwelling time of the Novel-S group was longer than that in the Normal-S group (10.7 ± 1.7 days vs 7.2 ± 0.9 days, p = 0.001) (Table 2).

The rate of removing stents by hand in the Novel-S group was lower than that in the Normal-S group (93.5% vs 100%, p = 0.004), and the group took a longer time to remove ureteral stents (65.3 vs 26.7 s, p = 0.001), with greater cost for stent removal (10.4 vs 0 CY), while the medical costs for complications and the overall costs postoperatively were significantly lower than those in the Normal-S group: 17.3 vs 55.8 CY, 27.7 vs 55.8 CY, respectively (Table 2).

## Discussion

Ureteral stents are a fundamental part of modern urologists’ armamentarium. Approximately 80% of urologists report leaving an indwelling ureteric stent after uncomplicated URS^4^. However, once the stent is inserted, subsequent withdrawal is inevitable. The traditional removal method is cystoscopy, which is time consuming and laborious and, more importantly, augments the pain and injury to the patient^5^.

Given that degradable stents are not applicable for short periods of time, many studies have attempted to remove stents without cystoscopy. However, removal by magnetic adhesion deserves further verification of its safety and efficiency^17^; removal by a crochet hook^18^, which is suitable for women, has a limited advantage because stent withdrawal by cystoscopy causes slight injury to women.

Among the methods of removing stents without cystoscopy, ureteric stents with extraction strings are the most widely applied^4,5,7,10,19^. Their advantages are as follows. First, the removal procedure was simple, and professionals were not required^5^. Second, no invasive operation was performed, causing slight injury and pain with stent removal^7,20^. Third, almost no additional expense was charged for stent removal, which benefited the patients^10^. This method was safety, without increasing the risk of urinary tract infection or stent-related urinary symptoms, but increased the risk of stent dislodgement, even for patients requiring short-term stent placement^10,13^. Two main reasons can be inferred: the first is the string length exposed to the outside of the urethra, and the second is the exposure time of the string. This study found that, when the length of the string equaled L_urethra_ + 2 cm, the string was only excluded from the outside of the urethra during the urination process in most cases, diminishing the exposure time significantly, which apparently decreased the risk of stent dislodgement, no stent dislodgement occurred in the Novel-S group. Besides, this method improved patients’ comfort degree and tolerability, patients in the group experienced more comfort and reported less symptom, showed a lower incidence of emergency room visits and later selection to remove the stent compared to that in the Normal-S group. These positive results indicated that this novel method could be applicable for patients requiring long-term stents and could benefit these patients.

Undeniably, this novel method lowered the success rate of removal by hand, and some patients required ureteroscopy to remove the stent, which increased the time needed for stent removal. However, it did not cause significant injury or pain in the patients, and the pain score in the Novel-S group was not higher than that in the Normal-S group. Indeed, these patients paid additional medical expenses for ureteroscopy; however, the cost was far less than that to treat the complications of stent dislodgement or UTIs, visiting the emergency room or clinic. Therefore, the overall medical cost postoperatively in the Normal-S group was significantly higher than that in the Novel-S group.

For this reason that the string was only excluded to the outside of the urethra during the urinating process, two causes can be identified: first, the distal end of the ureteral stent moved forward to the internal orifice of the urethra under the force of current during urination; and second, the stent itself descended with the bladder shrinking in urination, causing the distal end to be closer to the internal orifice. These reasons together resulted in the exclusion of the string during urination. Later, the string was pulled into the urethra again when the distal curve of the stent crimped back to normal after urination.

Of the 108 patients in the Novel-S group, 7 patients could not discharge the extraction string outside the urethra, and 9 patients had the string outside the urethra all the time. The main reasons were related to the shortness or overlength of the string. Measuring precision and individual variation in the urethra might be the main causes through analysis.

Some limitations existed in this research. First, although no dislodgement was observed in the study, it was unclear whether this method could avoid dislodgement for patients with long-term stent indwelling, it was not clear whether this method was applicable for female patients either. Further research will be conducted. Second, whether L_urethra_ + 2 cm was the most appropriate length was not clear, and perhaps L_urethra_ + 3 cm or another length was more suitable, in addition, maybe measuring the urethra length by ureteroscope is not the only way can be applied to determine the string length, cutting the string just beyond the external urethral meatus will also do the task accurately if some equation or formula can be elicited. Third, it is a retrospective analysis, not a randomized trial, and which method to choose was primarily based on surgeon preferences. It remains unclear whether this decision has been biased by other considerations.

## Conclusions

An appropriate length of the extraction string determined by measuring the personal urethra length shortened the exposure time of the string outside the urethra significantly for men, thereby, improved stenting comfort and patients’ tolerability of stent dwelling, decreased the risk of ureteral stent dislodgement and complications, and lowered the medical cost. The string length was recommended to be L_urethra_ + 2 cm. This method improved the safety of the ureteral stent with string, and is also likely to be applicable for patients requiring long-term stents, it merits widespread application.

## Supplementary Information

Supplementary Information 1.

Supplementary Information 2.

Supplementary Information 3.

Supplementary Information 4.

## Data Availability

All data analyzed during this study are included in this paper.
