# Supplementary figures and images for: Novel method to decrease the exposure time of the extraction string of the ureteral stent and its efficiency and safety verification in the clinic
Source: Sci Rep. 2021 Nov 16;11:22358. doi: 10.1038/s41598-021-01821-2 (PMC8595459; doi:10.1038/s41598-021-01821-2)

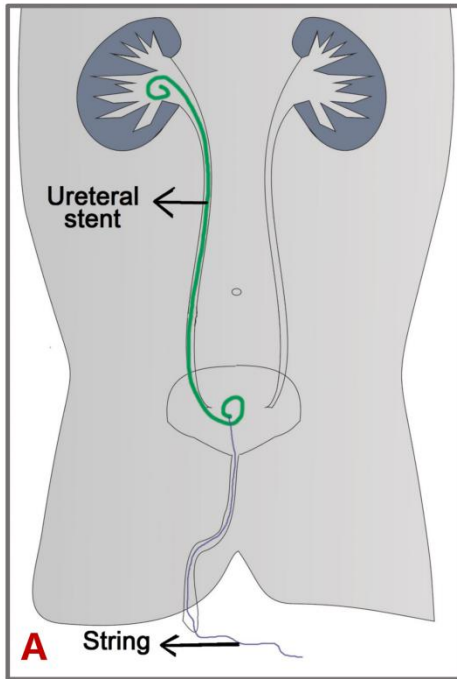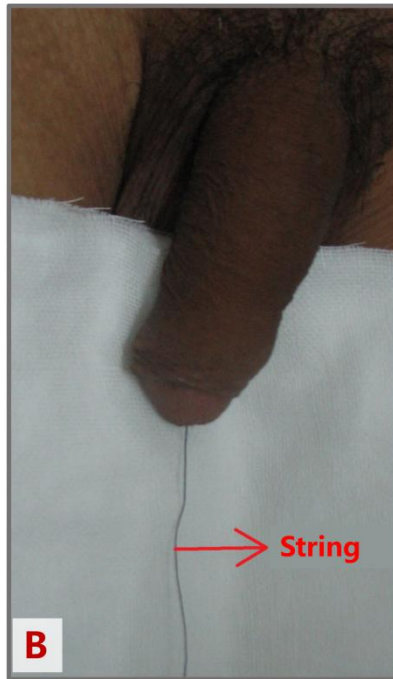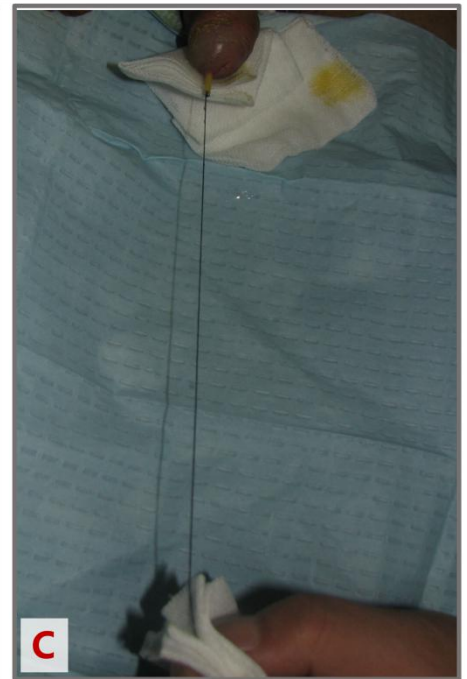

Supplementary material 1. Stent removal with the extraction string discharged.

Supplement: Supplementary file 1 — Supplementary Information 1. [file 41598_2021_1821_MOESM1_ESM.pdf]

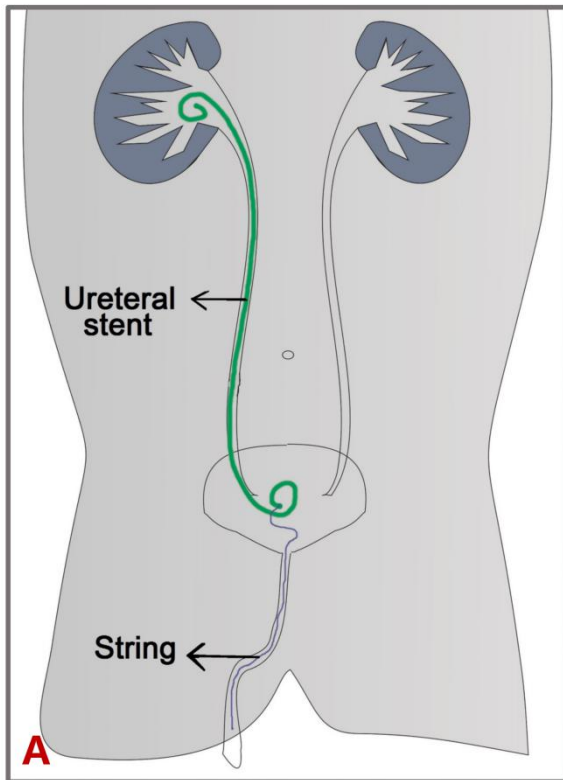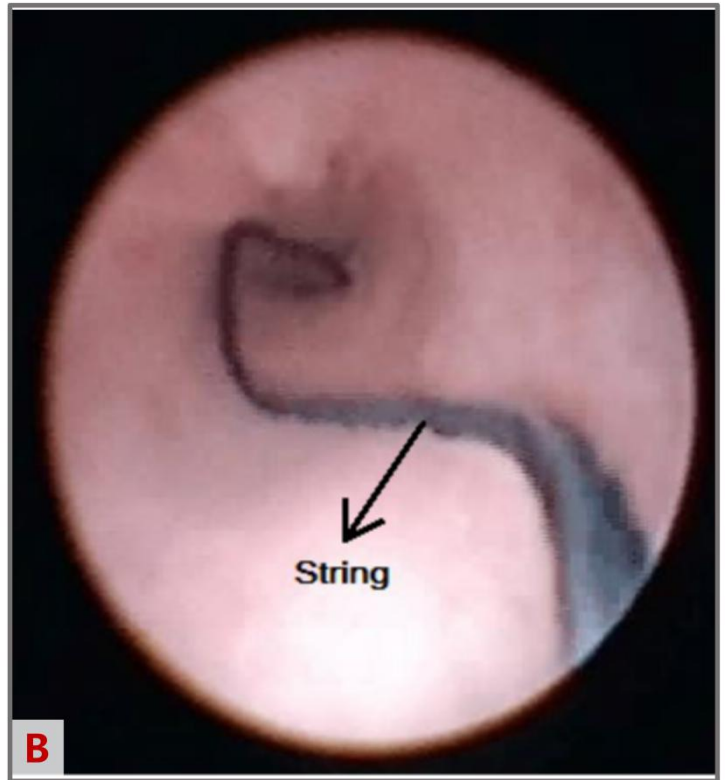

Supplementary material 3. Extraction string was only discharged into the anterior urethra.

Supplement: Supplementary file 3 — Supplementary Information 3. [file 41598_2021_1821_MOESM3_ESM.pdf]
