# Supplementary material for: Novel method to decrease the exposure time of the extraction string of the ureteral stent and its efficiency and safety verification in the clinic
Source: Sci Rep. 2021 Nov 16;11:22358. doi: 10.1038/s41598-021-01821-2 (PMC8595459; doi:10.1038/s41598-021-01821-2)
Supplement: Supplementary file 2 — Supplementary Information 2. [file 41598_2021_1821_MOESM2_ESM.pdf]

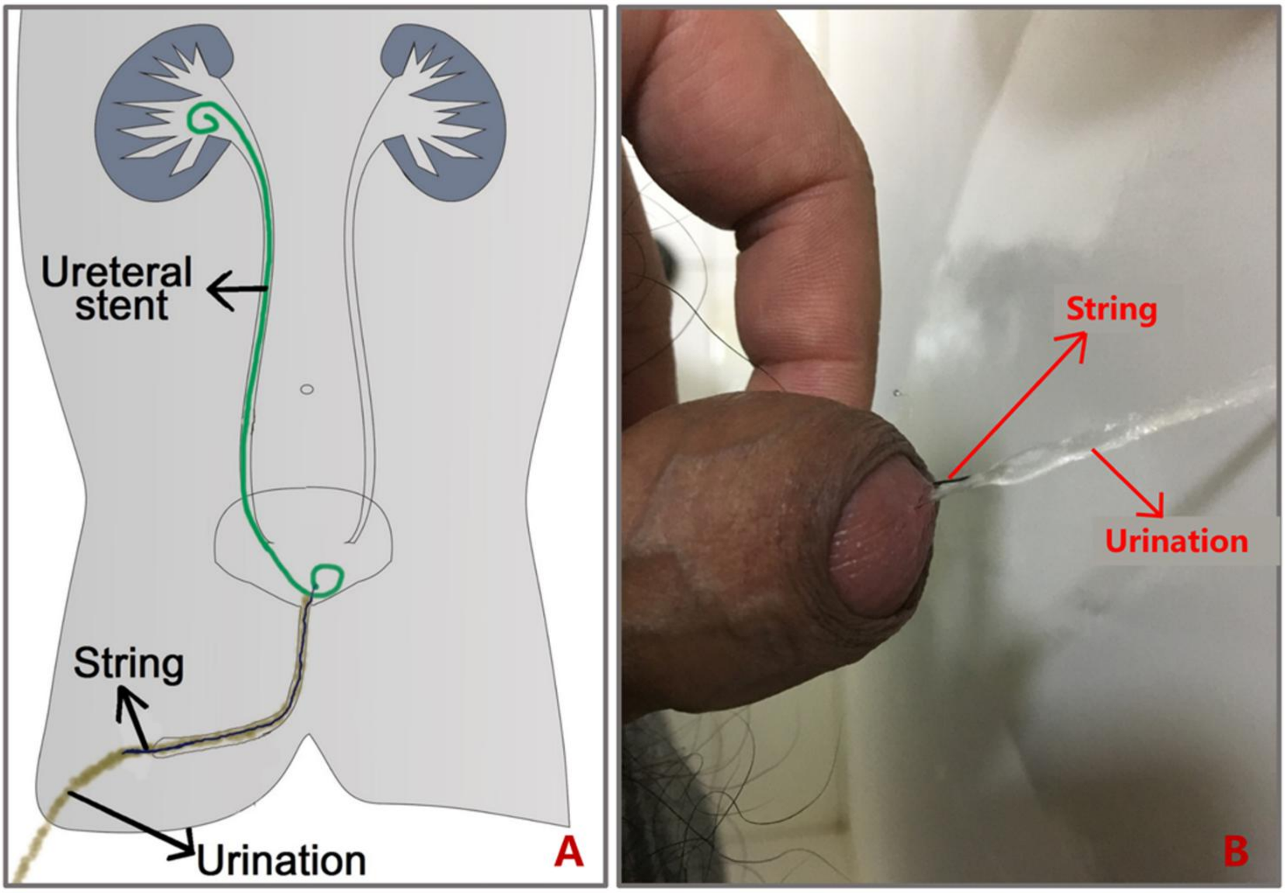

Supplementary material 2. Extraction string was discharged outside the urinary meatus just during urination.
