# Supplementary material for: Novel method to decrease the exposure time of the extraction string of the ureteral stent and its efficiency and safety verification in the clinic
Source: Sci Rep. 2021 Nov 16;11:22358. doi: 10.1038/s41598-021-01821-2 (PMC8595459; doi:10.1038/s41598-021-01821-2)
Supplement: Supplementary file 4 — Supplementary Information 4. [file 41598_2021_1821_MOESM4_ESM.pdf]

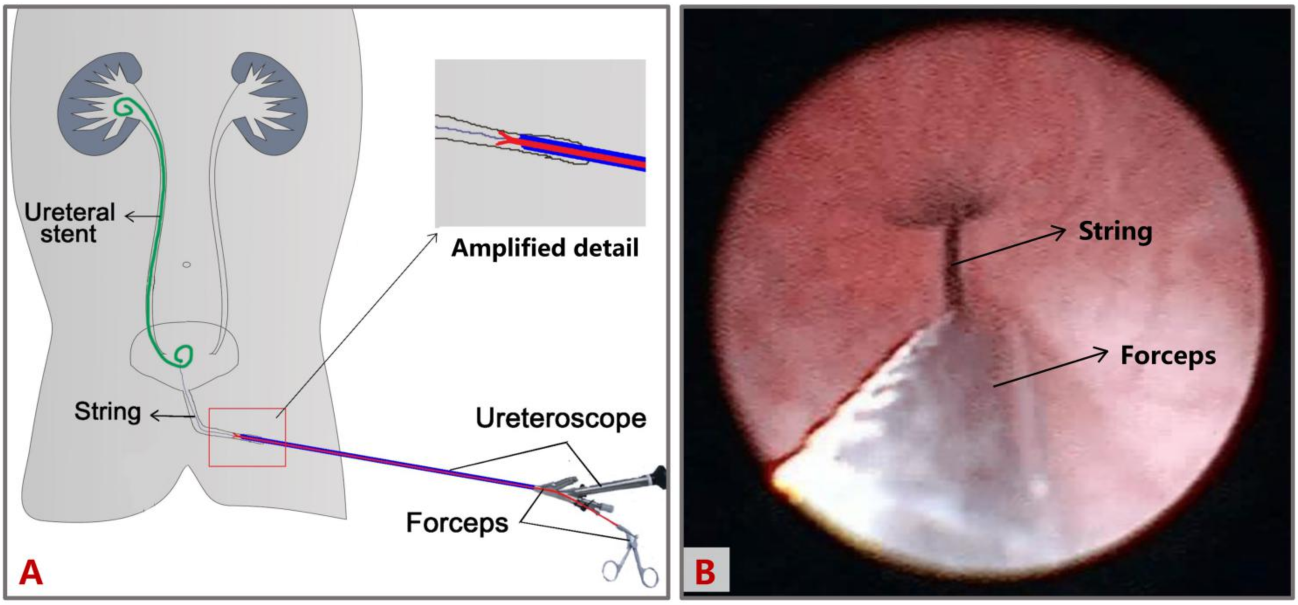

Supplementary material 4. Stent removal in the anterior urethra by the extraction string during ureteroscopy.
